# No positive effect of autologous platelet gel after total knee arthroplasty: A double-blind randomized controlled trial: 102 patients with a 3-month follow-up

**DOI:** 10.3109/17453670903350081

**Published:** 2009-10-01

**Authors:** Joost C Peerbooms, Gideon S de Wolf, Joost W Colaris, Daniël J Bruijn, Jan A N Verhaar

**Affiliations:** ^1^Department of Orthopaedic Surgery, HAGA Hospital, The Haguethe Netherlands; ^2^Department of Clinical Epidemiology and Biostatistics, Academic Medical CentreAmsterdamthe Netherlands; ^3^Department of Orthopaedic Surgery, Erasmus Medical CentreRotterdamthe Netherlands

## Abstract

**Background and purpose** Activated platelets release a cocktail of growth factors, some of which are thought to stimulate repair. We investigated whether the use of autologous platelet gel (PG) in total knee arthroplasty (TKA) would improve wound healing and knee function, and reduce blood loss and the use of analgesics.

**Patients and methods** 102 patients undergoing TKA were randomly assigned to a PG group (n = 50) or to a control (C) group (n = 52). The primary analysis was based on 73 participants (PG: 32; C: 41) with comparison of postoperative wound scores, VAS, WOMAC, knee function, use of analgesics, and the pre- and postoperative hemoglobin values after a follow-up of 3 months. 29 participants were excluded due to insufficient data.

**Results** The characteristics of the protocol-compliant patients were similar to those of the patients who were excluded. Analysis was per protocol and focused on the remaining 73 patients. At baseline and after 3 months of follow-up, there were no statistically significant differences between both groups regarding age, height, weight, sex, side of operation, platelet count, hemoglobin values, severity of complaints (WOMAC), and level of pain.

**Interpretation** In our patients undergoing TKA, application of PG to the wound site did not promote wound healing. Also, we found that PG had no effect on pain, knee function, or hemoglobin values.

## Introduction

Identification of methods to enhance or accelerate wound healing may be important, especially in high-risk patients (e.g. with type 1 diabetes mellitus, tobacco use, or previously irradiated tissue). The requirement for growth factors within the wound-healing cascade has been confirmed (Krupski et al. 1991, Hosgood 1993, Adler and Kent 2002). In a canine model, treatment with autologous blood platelet concentrate was found to enhance and accelerate early wound healing (Sclafani et al. 2005). In humans, autologous blood platelet concentrate was shown to increase bone formation in maxillofacial surgery (Marx et al. 1998). Since this latter result was regarded as a general stimulation of repair rather than a specific increase in bone formation, we investigated in a double-blind randomized trial whether the application of a platelet concentrate (in spray form) might improve the repair of wounds after TKA. The primary outcome parameter was wound healing, but we also studied the effects on knee function, use of analgesics, and hemoglobin values.

## Patients and methods

This double-blind, randomized study involved 102 consecutive patients scheduled for primary unilateral TKA for osteoarthritis between June 2005 and March 2007.

All procedures took place in a training hospital using the same surgical procedure performed by an orthopedic consultant or a supervised senior orthopedic resident. There was no age limit for inclusion. Criteria for participation included pain and radiographic knee osteoarthritis. Exclusion criteria were: platelet count ≤ 150 × 10^9^/L, hemoglobin level ≤ 6.5 mmol/L, BMI > 33, and systemic disorders such as diabetes, rheumatoid arthritis, and hepatitis.

We tested the hypothesis that the application of a platelet concentrate (in spray form) would improve repair of wounds after TKA. The primary endpoint was wound healing. In addition, we determined whether there were any effects on knee function, use of analgesics, and hemoglobin values.

### Randomization

Block randomization of the patients was performed after they were deemed eligible and had provided informed consent. Patients were randomly allocated to the PG arm or to the C arm. Treatment assignments (placed in sequentially numbered opaque envelopes) were done by the trial managers, who also arranged the facilities needed for the procedure.

### Surgical procedure

The medial parapatellar approach was used, averting the patella laterally. A tourniquet was used. In all cases, a cemented posterior cruciate retaining prosthesis (AGC; Biomet Biologics, Warsaw, IN) was used. After implantation of the components, the tourniquet was deflated and primary hemostasis was achieved. Before closure of the wound layers, the soft tissues and knee joint were rinsed with saline solution to remove all debris. After closure of the joint capsule, the subcutaneous tissues of the patients randomized to receive PG were sprayed with the platelet-poor plasma (PPP) fraction (approx. 10 mL) and the skin was closed with staplers. We did not use a deep or subcutaneous drain. In all patients, the knee incision was dressed postoperatively with compression bandages and rehabilitation was started on the day after surgery. In this fashion, patients, nurses, and physiotherapist were all blinded as to which procedure was used.

### Platelet-rich plasma preparation

In the group randomized to receive PRP, the patient's own platelets were collected using the GPS System (Biomet Inc.). This device uses a desktop-size centrifuge with disposable cylinders to isolate the platelet-rich fraction from a small volume of the patient's anticoagulated blood drawn at the time of the procedure.

First, a 60-mL syringe was filled with 7 mL of anticoagulant citrate phosphate dextrose formula A and 53 mL of whole blood was drawn via an intravenous catheter in the medial cubital vein using a 17-G needle. Proper mixing with the anticoagulant was done by inverting the syringe 8 times. The platelet-rich fraction was prepared according to the instructions for the use of the GPS system. In brief, blood was drawn into a 60-mL bowl of the blood cell separator and centrifuged for 15 min at a rate of 3,200 RPM for sequestration. Approximately 6 mL of PRP was obtained from each patient. Autologous thrombin was isolated from PRP and 0.17 mL of 10% calcium chloride to antagonize the anticoagulant in the donated blood. The platelets were activated by addition of calcified thrombin. The total time from drawing of blood to injection in the patients was about 90 min. No specialized equipment other than the centrifuge to process the GPS disposable was required. A person who was certified in blood management performed all the procedures under sterile conditions.

### Injection technique

Using a spray tip at a distance of 10–15 cm with the knee flexed at 90 degrees, which exposes the knee cavity, 6 mL PRP was applied to the dried wound site (synovium and bony cutting edges of femur and tibia); thereafter, the wound was closed in layers. After closure of the joint capsule, the subcutaneous tissues of the patients randomized to receive PG were sprayed with the platelet-poor plasma (PPP) fraction (10 mL approximately).

### Rehabilitation

Postoperative pain relief was achieved using a standard protocol: paracetamol (3 g daily) and diclofenac (50 mg 3 times daily), with pantoprazol (40 mg daily) for protection against ulcers. All patients received thrombosis prophylaxis from a subcutaneous injection of 0.3 mL low-molecular-weight heparin daily before the operation, and this was continued until a sufficient effect of oral anticoagulants (acenocoumarol) was achieved. The oral anticoagulants were used up to 12 weeks postoperatively. Rehabilitation, which was started on the day after surgery using crutches, was according to the Joint Care program (Biomet). The physiotherapist was also blinded as to which procedure was used.

### Wound score form

A wound score form was used for scoring wound healing (Table 1; see supplementary data). A pilot study showed the wound score form to be sufficiently reliable (K = 0.8, unpublished data). The score ranged from 0 to 100, with 0 representing a dry wound without any sign of infection, and 100 representing wound leakage with signs of infection. Points relating to changes of wound dressings (question 2) were multiplied by the score from the type of wound dressing (question 1: ×1 or ×2)]. Questions 3 and 4 were indicators of wound leakage, questions 5 and 7 were indicators of the type of leakage, and question 6 addressed wound infection parameters. The wound scores were measured by a trained orthopedics resident (medical ward) who did not know whether PG had been used.

### Statistics

There is little published information on whether the effect of application of a platelet concentrate can improve the repair of wounds after TKA. The purpose of this study was to investigate whether the application of a platelet concentrate would reduce 25% of the wound leakage. Wound leakage was regarded as a binary result (leakage or no leakage).

With a bilateral alpha of 0.05 and a power of 80%, 43 patients were required in each group to show a significant difference (α = 0.05, β = 0.8, 2n = 86). This difference is based on a study of Gardner et al. (2006).

Wound scores and function scores were measured on days 3–5, and at the regular control every 2 weeks. The function scores were also measured at 6 and 12 weeks postoperatively. For the purposes of analysis, the wound scores were dichotomized according to either “wound closure” (with a score of 0; no leakage or signs of infection) or “wound leakage” (scores > 0). Absolute differences in the rate of wound closure with corresponding confidence intervals were computed according to Altman (1991). Wound closure was analyzed with Chi-square test between the 2 groups for each day.

Hemoglobin values were measured pre- and postoperatively. The drop in hemoglobin was analyzed using unpaired Student's t-test.

Visual analog scale (VAS) for pain at rest and pain during walking was measured at intake and 6 weeks and 12 weeks postoperatively. The analog scores 0–10 mm (negligible), 10–30 mm (mild), 30–50 mm (painful), 50–80 mm (moderate), and 80–100 (severe) were regarded as ordinal categories. Analysis was focused on the changes between measurement points in time, using Mann-Whitney U tests. Postoperative frequency of use of analgesics was scored on a 5-point scale (never to always) preoperatively, and 6 weeks and 3 months postoperatively. Changes between measurement points in time were analyzed using Mann-Whitney tests.

The range of motion of the operated knee was analyzed on the second day, the third day, and the fourth day, and also 2 weeks, 6 weeks, and 3 months postoperatively using ANOVA for repeated measurements. WOMAC function scores were measured preoperatively, and 6 and 12 weeks postoperatively. Analysis focused on changes between measurement points in time, using Mann-Whitney U tests. A trained medical person who was blinded as to the treatment group measured all scores.

All data analyses were intended to be carried out according to a pre-established plan of analysis based on the principle of intention to treat. The significance level was set at p = 0.05. SPSS software version 16.0 for Windows was used.

### Ethics

All patients had to be able to read and understand the protocol and the informed consent document. The Medical-Ethical Commission (METC) and the National and Institutional Review Board approved the study (METC protocol number 04-17; date of approval October 27, 2004). The trial was performed in compliance with the Helsinki Declaration (2000) and Good Clinical Practice (1997).

## Results

From June 2005 to March 2007, a total of 111 patients with an indication for TKA were included in the study. 9 patients had to be excluded because of inclusion errors. Analysis was per protocol and focused on the remaining 102 patients. This was done according to a pre-established analysis plan based on the principle of intention to treat: 50 TKA patients were treated with PG and 52 were treated without PG. Patient characteristics at baseline were similar in the 2 groups (Table 2).

**Table 2.** Patient characteristics at inclusion

	PG group	Control group
No. of patients	50	52
No. of males	13 (26%)	11 (21%)
Age, mean (SD)	77 (4.4)	78 (5.1)
Length in cm, mean (SD)	168 (9.1)	168 (8.1)
Weight in kg, mean (SD)	83 (16)	79 (12)
Platelet count in 10^9^/L, mean (SD)	253 (63)	273 (64)
Hb in mmol/L, mean (SD)	8.6 (0.9)	8.5 (0.7)
Right side	29 (58%)	30 (59%)
WOMAC score, median (range)	49 (8–78)	43 (14–74)
Pain at rest, median (range)	3 (1–5)	3 (1–5)
Pain during activity, median (range)	4 (1–5)	4 (1–5)

Due to a reorganization of the patient's ward, however, no or partial measurements were recorded for a number of the study patients during their hospital stay. Missing data were attributed based on the “last known result carried forward” principle.

The characteristics of the patients who were fully recorded were similar to those of the other patients (Table 3). Eventually, full data were recorded for 32 patients in the PG group and 41 patients in the C group. Analysis for possible bias caused by missing data showed no differences between the resulting groups at baseline (Table 3, Figure).

**Figure:**
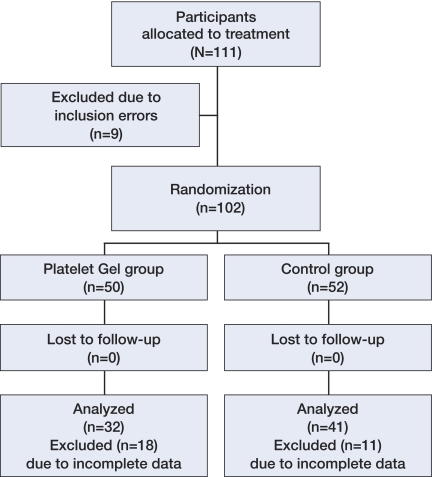
Flow chart of the patients.

**Table 3.** Data of the patients with and without in-hospital recorded data

	Complete recovery	Incomplete recovery	p-value	PG group	Control group	p-value
No. of patients	73	29		32	41	
No. of males	14 (19%)	10 (34%)	0.1	6 (19%)	8 (20%)	1
Age, mean (SD)	77 (4.8)	77 (4.8)	0.9	76 (4.1)	78 (5.2)	0.1
Length in cm, mean (SD)	167 (7.6)	169 (10.7)	0.3	166 (6.5)	168 (8.3)	0.4
Weight in kg, mean (SD)	80 (14)	81 (15)	0.8	81 (17)	80 (12)	0.6
Platelet count in 10^9^/L, mean (SD)	269 (66)	249 (58)	0.2	261 (68)	275 (64)	0.4
Hb in mmol/L, mean (SD)	8.6 (0.8)	8.6 (0.9)	0.9	8.6 (0.8)	8.5 (0.7)	0.3
Right side	43 (59%)	16 (57%)	1	16 (50%)	27 (66%)	0.2
WOMAC score, median (range)	45 (8–76)	41 (11–76)	0.4	50 (8–76)	43 (14–74)	0.6
Pain at rest, median (range)	3 (1–5)	3 (1–5)	0.8	4 (1–5)	3 (1–5)	0.6
Pain during activity, median (range)	4 (1–5)	4 (2–5)	0.9	4 (1–5)	4 (1–5)	0.3

### Primary endpoint (wound healing)

Both groups had normal wound healing (Table 4). No clinically or statistically significant differences in wound closure were noted between the 2 groups during the hospital stay (third day, 95% CI: 11% to 25% difference; fourth day, 95% CI: 30% to 10% difference). At 2 weeks, significantly more patients in the control group had total wound closure than in the PG group (p = 0.02) (95% CI: –41% to –7% difference).

**Table 4.** Data on the platelet gel group (PG) and the control group

	PG group	Control group	95% CI	p-value
Wound closure )	n = 32	n = 41		
Second day postoperatively	0	0		–
Third day postoperatively	7	6	7% (–11% to 25%)	0.5
Fourth day postoperatively	7	13	–9% (–30% to 10%)	0.4
Wound closure	n = 36	n = 46		
2 weeks postoperatively	4	16	–24% (–41% to 7%)	0.02
Drop in Hb, mean (SD)	n = 50	n = 52		
mmol/L	–1.58 (0.63)	–1.75 (0.58)	0.16 (–0.07 to 0.4)	0.2
Pain at rest, median (range)	n = 50	n = 52		
At inclusion	3 (1–5)	3 (1–5)		0.8
6 weeks	2 (1–5)	2 (1–5)		0.08
3 months	2 (1–5)	2 (1–5)		0.8
Pain during walking, median (range)	n = 50	n = 52		
At inclusion	4 (1–5)	4 (1–5)		0.4
6 weeks	2 (1–5)	2 (1–5)		0.07
3 months	2 (1–5)	2 (1–5)		0.9
Use of pain medication, median (range)	n = 50	n = 52		
At inclusion	2 (1–5)	2 (1–5)		0.5
6 weeks	2 (1–5)	2 (1–5)		0.9
3 months	2 (1–5)	2 (1–5)		0.1
WOMAC score, mean (range)	n = 50	n = 52		
At inclusion	45 (8–76)	44 (14–74)		
6 weeks	26 (3–76)	24 (0–65)	0 (–8 to 8)	0.7
3 months	25 (0–76)	21 (0–66)	–3 (–6 to 1)	0.4
ROM, mean (SD)	n = 32	n = 36		
Second day postoperatively	53 (14)	50 (17)		
Third day postoperatively	68 (13)	65 (16)		
Fourth day postoperatively	72 (13)	73 (14)		0.7
ROM, mean (SD)	n = 34	n = 45		
2 weeks postoperatively	91 (13)	89 (13)		
6 weeks postoperatively	99 (11)	100 (13)		
3 months postoperatively	102 (12)	101 (12)		0.9

### Secondary endpoints

There was a difference in the postoperative drop in levels of hemoglobin between the 2 groups (between-group difference: 0.16 mmol/L, p = 0.2; 95% CI: –0.07 to 0.4 difference) (Table 4).

Reported pain was reduced from (moderately) painful to mild pain 6 weeks postoperatively. There was a trend of greater pain reduction (both at rest and while walking) at 6 weeks postoperatively for the C group.

The median frequency of use of medication was “sometimes”, and this remained the median answer thoughout the study period. There was no difference in reduction of use of pain medication between the PG group and the C group, either at 6 weeks postoperatively (p = 0.9) or at 3 months (p = 0.1).

As expected, during hospitalization the range of motion of the operated knee increased from 50 degrees 2 days after surgery to 75 degrees at discharge from hospital. During these days, no benefit was seen for the PG group (2-way ANOVA, p = 0.7). At 2 weeks, 6 weeks, and up to 3 months of follow-up, no differences were seen between the two groups (p = 0.9).

At 6 weeks postoperatively, the self-rated knee function (WOMAC score) had increased by 20 points, but the recovery rate between the two groups was similar (Mann-Whitney U test, p = 0.7; 95% CI: –8 to 8 difference); similarly, there were no differences between groups at the 3-month follow-up (95% CI: –6 to 1 difference).

### Complications

After discharge from hospital, superficial wound infections occurred in 2 patients (1 in each group, both of which were caused by coagulase-negative *Staphylococcus*); these infections were treated successfully with antibiotics. No deep infections were seen.

## Discussion

In this randomized study, we found no effect on wound healing of platelet gel (PG) used after TKA.

PG is promoted as an ideal autologous biological blood-derived product that can be exogenously applied to various tissues where it releases high concentrations of platelet growth factors that enhance wound healing. In addition, PG has antimicrobial properties that may contribute to the prevention of infections (Everts et al. 2007). When platelets become activated, growth factors are released and they initiate the body's natural healing response.

The actual quantity of platelets needed to achieve an improved outcome when PG is used is still unknown. Marx et al. (1998) found that a 3–4 times higher platelet count improved the mandibular continuity defects. The GPS system that we used produces a 6–8 times higher platelet count. Much higher concentrations might have an inhibitory effect (Weibrich et al. 2004). The activator for the platelets we used was a mixture of thrombin and calcium chloride. After combining these substances, platelet-rich gel is formed and numerous regulatory molecules and antimicrobial proteins are released to the injury site (Wrotniak et al. 2007). Thrombin derived from bovine plasma is used in the USA, despite the fact that bovine thrombin was associated years ago with the development of antibodies to thrombin and factor V, which had led to recurrent bleeding in patients who were exposed (Zehnder and Leung 1990). Alternatively, the platelets can be activated by autologous thrombin, produced with commercially available thrombin kits (Everts et al. 2006 b, c). Tsay et al. (2005) described the use of a synthetic peptide that mimics thrombin, known as peptide-6 SFLLRN (TRAP).

Using the GPS system, the patient's own platelets (which travel through the bloodstream) can be collected into a highly concentrated formula.

We found a slight difference in the drop in hemoglobin: 0.16 mmol/L. This was 10% of the total drop. The mean hemoglobin before operation was 8.6 mmol/L. After operation, the hemoglobin in the PG group dropped to 7.1 mmol/L and in the control group it dropped to 7.0 mmol/L. This finding contrasts with an earlier report, which might be explained by differences in the technique and methodology. For example, Everts et al. (2006a) used a PG and fibrin sealant technique, a preparation that differs from our technique. Moreover, their trial included more patients and the hemoglobin values were scored not only on the first postoperative day (as in our study) but also on days 2–4 after surgery, and again on the day of hospital discharge. Everts et al. only scored function during the first 5 days and on the day of discharge, whereas we scored function on the first 4 days, and at 2, 6, and 12 weeks postoperatively.

The beneficial effects of concentrated growth factors are said to reduce wound leakage by 25%, minimizing the need for postoperative blood transfusion, reducing the risk of postoperative infections, and promoting faster functional rehabilitation with less pain (Floryan and Berghoff 2004, Gardner et al. 2006). Most reports on PG have involved its use for healing chronic wounds (Hom and Maisel 1992, Hosgood 1993, Wang et al. 1996, Powell et al. 2001, Crovetti et al. 2004). To our knowledge, no blind randomized study has been performed previously.

The type of wound dressing is also important. It has been shown that with the use of occlusive dressings, both re-epithelialization and subsequent collagen synthesis are 2–6 times faster than they are in wounds exposed to air. At the cellular level, dressings assist wound healing by creating a hypoxic wound environment in which fibroblasts proliferate and angiogenesis occurs more rapidly (Fletcher et al. 2007). The proper timing of dressing removal remains a controversial subject. Studies on clean, and clean contaminated, wounds have shown no difference in infection rates between whether the dressing was removed on the first postoperative day or at the time of suture removal (Chrintzet al. 1989, Meylan and Tschantz 2001). In our patients, all wounds were dressed with sealed bandages directly after surgery and undressed on the second day after surgery; no beneficial effect of PG was seen. The use of PG has shown good results in wounds that are difficult to heal compared to normal wound treatment (Margolis et al. 2001, Henderson et al. 2003, Mazzuco et al. 2004), but exogenously applied platelets have no hemostatic effect. They have poor tensile strength to accomplish sealing of the wound. Altmeppen et al. (2004) showed that an autologous platelet-enriched plasma cannot be used as a glue in the common sense to seal stitches or prostheses. Platelet gels, however, have a high concentration of platelets that release the bioactive proteins and growth factors that are necessary to initiate and accelerate tissue repair and enhance dermal and epidermal regeneration.

We used a specially designed wound scoring system; to our knowledge, no measure of wound leakage has previously been described for this type of study.

Several of the patients had incomplete datasets. This was because of integration of 2 hospitals into one during the second half of the trial; we underestimated the deleterious effect of this reorganization on the quality of data collection at the ward level. There is no clear explanation for the difference between the rate at which patients from both groups were not recorded; we can only assume that this was coincidental. Between-group analysis of patient characteristics at baseline did not show any statistically significant differences in the patients with or without complete data recoding. Due to this dropout of data, we had a severe loss of statistical power with regard to our primary outcome measure (wound closure). Our results regarding wound closure do, however, follow a similar trend to those of all secondary outcome measures and are in accordance with our clinical observations, thus strengthening our conclusion. Another limitation of our study was that we did not investigate the effect of varying platelet and fibrinogen concentrations.
